# Human Milk Fortification in Very Preterm Infants in China: A Multicenter Survey

**DOI:** 10.3389/fped.2022.795222

**Published:** 2022-02-23

**Authors:** Rong Lin, Wei Shen, Fan Wu, Jian Mao, Ling Liu, Yanmei Chang, Rong Zhang, Xiuzhen Ye, Yinping Qiu, Li Ma, Rui Cheng, Hui Wu, Dongmei Chen, Zhi Zheng, Xinzhu Lin, Xiaomei Tong, the National Multicenter EUGR Collaborative Group

**Affiliations:** ^1^Department of Neonatology, Women and Children's Hospital, School of Medicine, Xiamen University, Xiamen, China; ^2^Xiamen Key Laboratory of Perinatal-Neonatal Infection, Xiamen, China; ^3^Department of Neonatology, The Third Affiliated Hospital of Guangzhou Medical University, Guangzhou, China; ^4^Department of Pediatrics, Shengjing Hospital of China Medical University, Shenyang, China; ^5^Department of Neonatology, Guiyang Maternal and Child Health Hospital Guiyang Children's Hospital, Guiyang, China; ^6^Department of Pediatrics, Peking University Third Hospital, Beijing, China; ^7^Department of Neonatology, Pediatric Hospital of Fudan University, Shanghai, China; ^8^Department of Neonatology, Guangdong Province Maternal and Children's Hospital, Guangzhou, China; ^9^Department of Neonatology, General Hospital of Ningxia Medical University, Yinchuan, China; ^10^Department of Neonatology, Children's Hospital of Hebei Province, Shijiazhuang, China; ^11^Department of Neonatology, Children's Hospital of Nanjing Medical University, Nanjing, China; ^12^Department of Neonatology, The First Hospital of Jilin University, Changchun, China; ^13^Department of Neonatology, Quanzhou Maternity and Children's Hospital, Quanzhou, China

**Keywords:** human milk fortifier, very preterm infants, extrauterine growth restriction, feeding intolerance, human milk

## Abstract

**Aim:**

To investigate the use of human milk fortifier (HMF) for very preterm infants (VPIs) and complications and nutritional status of VPIs due to various breast milk enhancement strategies among the Chinese population.

**Methods:**

VPIs with birth weight < 1,800 g and wholly or predominantly breastfed were assigned to the following fortification groups: no HMF, early HMF (adding HMF at an enteral volume of ≤ 80 ml·kg^−1^·day^−1^), middle HMF (adding HMF at an enteral volume of 80–100 ml·kg^−1^·day^−1^), and late HMF (adding HMF at an enteral volume of ≥100 ml·kg^−1^·day^−1^). The growth status and complications for various groups were evaluated.

**Results:**

We enrolled 985 VPIs, of which 847 VPIs (86.0%) received HMF, whereas 138 VPIs (14.0%) did not. The number of VPIs in the early, middle, and late fortification groups were 89 (9.0%), 252 (25.6%), and 506 (51.4%), respectively. The complete fortification of the early, middle, and late fortification groups was achieved in 13.2 ± 11.0, 13.8 ± 11.7, and 12.3 ± 13.0 days, respectively, without significant differences (*p* > 0.05). The groups did not exhibit significant differences in the incidence of feeding intolerance, necrotizing enterocolitis (Bell stage ≥ 2), late-onset sepsis, and metabolic bone diseases (*p* > 0.05). The middle fortification groups exhibited the fastest growth velocity and the least dramatic decrease in the *Z*-score of weight and length, and the lowest incidence of EUGR (35.7%), whereas the “no HMF” groups exhibited the slowest growth velocity and the largest decline in the *Z*-score, and the highest incidence of EUGR (61.6%).

**Conclusions:**

The usage rate of HMF was relatively low among Chinese VPIs, fortification often occurred in the late feeding stage, and the time to reach complete fortification was long. Adding HMF and different breast milk enhancement strategies did not increase the incidence of feeding intolerance and necrotizing enterocolitis. The enteral volume of 80–100 ml·kg^−1^·day^−1^ with HMF addition led to increased growth in the weight and length and lower EUGR incidence, indicating that the addition of HMF at the specific feeding volume might be the best practice for promoting growth.

## Introduction

Human milk is the best source of nutrition for all infants, especially for preterm infants, as it better compensates for the immature immune, vascular, and neurological systems. Breastfeeding assists preterm infants in achieving full enteral nutrition right after birth, reduces in-hospital infections and necrotizing enterocolitis (NEC) occurrence, and enhances nervous system development (1). However, from a nutritional point of view, human milk alone cannot provide sufficient energy and nutrition for preterm infants. Human milk intake as high as 250–350 ml/kg/day may theoretically cover protein needs for preterm infants, but a high intake does not correct the suboptimal protein-to-energy ratio with the resulting risk of excessive fat deposition (2). Breastfeeding without human milk fortifier (HMF) results in the development of metabolic bone diseases and other complications. To prevent nutritional insufficiencies related to human milk while taking advantage of its biological properties, HMF are used for preterm infants (3).

Adding HMF to human milk is necessary to provide additional calories, protein, minerals, and vitamins to premature infants. Fortification of human milk can help to reduce the gaps in meeting nutrient needs and the incidence of extrauterine growth restriction (EUGR), and promotes bone mineralization and linear growth (3). Breast milk fortification has desirable effects on neural development of premature infants, decreasing the incidence of NEC and sepsis in comparison with those who were fed without fortification (4). The use of HMF is recommended, especially in premature infants weighing <2,000 g. The practice of timing of the fortification of human milk varies because of concerns about immature gut mucosa and motility in infants. Clinicians are sometimes concerned that addition of fortifiers may induce feeding intolerance and delay achieving full volume enteral feeds and optimal nutrition. Early fortification provides several benefits to infants such as provision of adequate calories, protein, and other nutrients compared with delayed fortification. While some studies have reported early and late onset of fortifiers having similar effects on infant's height, weight, and head circumference (5), others have suggested that early oral feeding along with fortifiers led to poorer weight gaining and no significant head circumference growth (6).

There was also no large sample study on the use of HMF for very preterm infants (VPIs) [<32 week's gestational age (GA)] in China. The purpose of the current multicenter prospective study was to analyze the impacts of various breast milk enhancement strategies on the complications and nutritional status of VPIs, providing the basis for nutritional strategy optimization.

## Materials and Methods

The Chinese Multicenter EUGR Collaborative Group was found in 2019, with the aim of investigating the incidence and related factors of EUGR in VPIs during hospitalization from different regions of China (Trial registration: chictr.org.cn, number: ChiCTR1900023 418). The clinical data of 2,561 cases of VPIs were prospective collected from 28 hospitals in 7 different regions of China between September 2019 and December 2020. We analyzed the data of HFM usage, complications, and growth status of the enrolled VPIs.

### Study Population

VPIs with birth weight (BW) < 1,800 g and wholly or predominantly breastfed (breastfeeding volume of ≥ 80% of the total enteral feeding volume) were eligible for the study. Infants with metabolic diseases or congenital malformations, those with a hospital stay of ≤14 days, and those transferred or who died before discharge were excluded.

A total of 985 VPIs were included and assigned into the following fortification groups: no HMF (138 cases), early (89 cases, adding HMF at an enteral volume of ≤ 80 ml·kg^−1^·day^−1^), middle (252 cases, adding HMF at an enteral volume of 80–100 ml·kg^−1^·day^−1^), and late (506 cases, adding HMF at an enteral volume of ≥100 ml·kg^−1^·day^−1^).

### Data Collection

The recorded demographic variables include sex, GA, Apgar score, twins and multiple births, intrauterine growth restriction (IUGR), complications, loss of birth weight, days to regain BW, duration of parenteral nutrition (PN), days to reach full feeding, days to full fortification, body weight, length, and head circumference (HC). Epi Data 3.1 software was used to collect the data and conduct consistency check by two clinic doctors.

### Feeding and Fortification

Enteral feedings were initiated with the attending physician's discretion as per a standardized feeding protocol, guiding the feeding method and increments of advancement. Feeding of the participants was started on the first day of life. After the first day, the feeding volume was increased by 10–20 ml·kg^−1^·day^−1^ to a maximum volume of 170–200 ml·kg^−1^·day^−1^. Full enteral feeding was defined as ≥150 ml·kg^−1^·day^−1^ of milk feeds actually administered for more than 24 h.

Human milk was provided by mothers or donors, and a commercial powdered HMF was used. Based on feeding tolerance, HMF started with a quarter or half dose of fortification on the first day and gradually increased until total fortification dosage was achieved. The dose of HMF required to achieve different degrees of fortification was confirmed as per the product instructions.

### Outcomes

Growth status was evaluated for VPIs, including the growth velocity of weight, length, and HC; change in *Z*-score of weight, length, and HC from birth until discharge; and the incidence of EUGR. During the hospital stay, the body weight is routinely measured by the attending nurses, using scales incorporated in incubators or external automatic scales. Weight gain velocity (g·kg^−1^·day^−1^) is calculated using an exponential model (7). Anthropometric measurements were done weekly until discharge. Length and HC gain were calculated as centimeters per week from birth till discharge. Length and HC were measured by an infantometer and a non-stretchable tape, respectively. *Z*-score and percentiles were calculated from the updated Fenton growth charts. We downloaded and used the Excel spreadsheets available on their website (8). The change in *Z*-score (Zdischarge–Zbirth) was calculated to illustrate the postnatal growth hospitalization. EUGR was defined as weight below 10th percentile at discharge (9).

The secondary outcome refers to the differences in complications, including feeding intolerance, NEC (Bell stage ≥ 2), bronchopulmonary dysplasia (BPD), late-onset sepsis, and metabolic bone diseases among the various groups. Incidence of complications was recorded as per standard definitions (4, 10–13).

### Statistical Analysis

The collected data were analyzed using SPSS for Windows, version 23.0 (SPSS Inc., Chicago, IL, USA). Continuous variables were reported as mean ± standard deviation (SD) or median and interquartile range (IQR), and categorical variables were reported as counts and percentages. We performed an analysis of variance (ANOVA) for normally distributed data, Kruskal–Wallis H rank-sum tests for skewed distributed data, and chi-square tests for categorical data, to compare differences among the four groups. If an overall test was statistically significant, a post-hoc analysis was conducted by using the Dunnett test or partitions of the χ^2^ method, to compare difference between the no-HMF group and the three fortification groups, respectively. Spearman correlation analysis was performed to determine the correlation between two variables. Multiple linear regression analysis was used to evaluate significant variables affecting the growth velocity of weight. OR with 95% CIs was calculated for all variables. The test level was set at α = 0.05. The cutoff for the significant difference was set at *p* < 0.05.

## Results

### Comparison of Baseline Characteristics

There were no significant differences in sex, donor human milk use, Apgar score, and incidence of IUGR (*p* > 0.05) among the four groups. However, significant differences were reported in the GA at birth and discharge, BW, birth length, birth HC, and hospital stay (*p* = 0.000) (Table 1). The early fortification group exhibited the lowest GA, weight, length, and HC at birth, the longest hospital stay, and the largest GA at discharge. At the same time, the no-HMF group had the largest GA, weight, length, and HC at birth, the shortest hospital stay, and the lowest GA at discharge (Table 1).

**Table 1:** Comparison of baseline characteristics.

**Group**	**No HMF**	**Early fortification**	**Middle fortification**	**Late fortification**	**p-value**
	**(*n* = 138)**	**(*n* = 89)**	**(*n* = 252)**	**(*n* = 506)**	
Male (*n*, %)	74 (53.6)	56 (62.9)	146 (57.9)	147 (55.1)	0.468
Donor human milk use (*n*, %)	4 (2.9)	1 (1.1)	10 (4.0)	21 (4.2)	0.519
BW (mean ± SD, g)	1,375 ± 232	1,204 ± 261^a^	1,255 ± 257 ^a^	1,293 ± 251^a^	0.000
Birth length (mean ± SD, cm)	38.7 ± 2.8	37.3 ± 3.0^a^	37.5 ± 3.3^a^	38.0 ± 3.1^a^	0.000
Birth HC (mean ± SD, cm)	27.4 ± 1.8	26.4 ± 2.1^a^	26.8 ± 1.8^a^	26.9 ± 1.9^a^	0.000
GA at birth [median (IQR), weeks]	30.4 (1.6)	29.1 (2.8)^a^	29.6 (2.6) ^a^	29.9 (2.3)^a^	0.000
GA at discharge (mean ± SD, weeks)	36.8 ± 1.8	37.8 ± 2.1^a^	37.1 ± 1.9	37.0 ± 1.9	0.000
Hospital stay [median (IQR), days]	45.5 (24)	60 (29)^a^	51 (23)^a^	48 (23)^a^	0.000
1 min Apgar score (mean ± SD)	7.51 ± 1.8	7.37 ± 1.8	7.32 ± 2.0	7.51 ± 1.9	0.569
5 min Apgar score (mean ± SD)	8.63 ± 1.2	8.48 ± 0.9	8.46 ± 1.1	8.62 ± 1.2	0.202
Twins and multiple births (*n*, %)	45 (32.6)	21 (17.3)	91 (36.1)	172 (34.0)	0.187
IUGR (*n*, %)	5 (3.6)	6 (6.7)	23 (8.8)	25 (4.9)	0.106

a *Significantly different between the No-HMF group and Fortification group*.

### Comparison of Complication Incidence

No significant differences were observed in the incidence of feeding intolerance, NEC (Bell stage ≥ 2), late-onset sepsis, and metabolic bone diseases (*p* > 0.05) (Table 2). The early fortification group exhibited the highest incidence of moderate to severe BPD, with statistically significant differences among the four groups (*p* = 0.000) (Table 2).

**Table 2:** Comparison of complication incidence.

**Group**	**No HMF**	**Early fortification**	**Middle fortification**	**Late fortification**	**p-value**
	**(*n* = 138)**	**(*n* = 89)**	**(*n* = 252)**	**(*n* = 506)**	
Feeding intolerance (*n*, %)	48 (34.8)	37 (41.6)	108 (42.9)	191 (37.7)	0.363
NEC [Bell stage ≥ 2, (*n*, %)]	9 (6.5)	2 (2.2)	7 (2.8)	19 (3.8)	0.246
Moderate to severe BPD (*n*, %)	14 (10.1)	36 (40.4)^a^	51 (28.8)^a^	76 (15.0)^a^	0.000
Late sepsis (*n*, %)	14 (7.4)	7 (7.9)	13 (5.2)	39 (7.7)	0.326
Metabolic bone diseases (*n*, %)	8 (5.8)	3 (3.4)	9 (3.6)	36 (4.7)	0.178

a *Significantly different between the No-HMF group and Fortification group*.

### Comparison of Nutritional Status

There were no significant differences in the loss of birth weight, days to regain BW, duration of parenteral nutrition (PN), days to reach full feeding, days to full fortification, and energy intake for the first week after birth (*p* > 0.05) among the four groups (Table 3).

**Table 3:** The comparison of nutritional status.

**Group**	**No HMF**	**Early fortification**	**Middle fortification**	**Late fortification**	**p-value**
	**(*n* = 138)**	**(*n* = 89)**	**(*n* = 252)**	**(*n* = 506)**	
Loss of birth weight (mean ± SD, %)	6.51 ± 4.7	6.24 ± 3.7	6.46 ± 4.4	6.82 ± 4.5	0.555
Days to regain BW (mean ± SD, days)	8.92 ± 4.7	9.33 ± 4.5	8.83 ± 3.8	9.05 ± 4.3	0.786
Duration of PN [Median (IQR), days]	21.0 (15)	24.0 (15.5)	22.0 (14)	20 (15)	0.062
Days to full enteral feeding (mean ± SD, days)	31.0 ± 13.9	32.1 ± 13.0	30.0 ± 12.5	30.3 ± 13.1	0.570
Energy intake for the first week (mean ± SD, kcal·kg^−1^·day^−1^)	485.0 ± 89.2	504.3 ± 120.4	498.6 ± 89.2	499.4 ± 104.9	0.430
Days to full fortification (mean ± SD, days)	-	13.2 ± 11.0	13.2 ± 11.0	13.2 ± 11.0	0.106
Discharge weight (mean ± SD, g)	2,178 ± 341	2,484 ± 456^a^	2,397 ± 394^a^	2,287 ± 402^a^	0.000
Discharge length (mean ± SD, cm)	44.5 ± 2.4	45.8 ± 2.2^a^	45.4 ± 2.4^a^	44.7 ± 2.7	0.000
Discharge HC (mean ± SD, cm)	31.4 ± 1.5	32.1 ± 2.3^a^	31.7 ± 1.4	31.4 ± 1.7	0.000
Weight growth velocity (mean ± SD, g·kg^−1^·day^−1^)	12.5 ± 3.8	14.1 ± 2.3^a^	15.0 ± 2.8^a^	13.7 ± 2.8^a^	0.000
Length growth velocity (mean ± SD, cm/week)	0.85 ± 0.4	0.97 ± 0.3	1.0 ± 0.4^a^	0.90 ± 0.4	0.000
HC growth velocity (mean ± SD, cm/week)	0.61 ± 0.3	0.64 ± 0.3	0.64 ± 0.2	0.62 ± 0.3	0.528
Change in *Z*-score of weight (mean ± SD)	−1.55 ± 0.7	−1.40 ± 0.8	−1.14 ± 0.7^a^	−1.36 ± 0.7^a^	0.000
Change in *Z*-score of length (mean ± SD)	−1.25 ± 1.2	−1.18 ± 1.2	−0.80 ± 1.2^a^	−1.12 ± 1.2	0.001
Change in *Z*-score of HC (mean ± SD)	−1.08 ± 1.2	−1.09 ± 1.7	−1.01 ± 1.1	−1.12 ± 1.3	0.709
EUGR (*n*, %)	85(61.6)	43(48.3)^a^	90(35.7)^a^	252(50.4)^a^	0.000

a *Significantly different between the No-HMF group and Fortification group*.

However, significant differences were observed in the weight, length, and HC at discharge (*p* = 0.000). The early fortification group and the no-HMF group had the largest and the lowest weight, length, and HC at discharge, respectively. Significant differences in the growth velocity and the change in *Z*-score of weight and length were noted (*p* = 0.000) (Table 3). The middle fortification group exhibited the fastest growth velocity and the least dramatic decrease in *Z*-score of weight and length, whereas the “no HMF” group had the slowest growth velocity and the largest decrease in *Z*-score of weight and length. No significant differences were observed in the growth velocity and change in the *Z*-score of HC (Table 3). The incidence of EUGR was significantly different among the four groups (*p* = 0.000), with the highest incidence in the no-HMF group (61.6%) and the lowest in the middle fortification group (35.7%) (Table 3).

### Correlation of Influencing Factors With Weight Growth Velocity

Results of Spearman's correlation analysis show that BW and hospital stay have a negative correlation with the weight growth velocity. Furthermore, change in *Z*-score of weight was positively correlated with the weight growth velocity (Table 4).

**Table 4:** Correlation of influencing factors with weight growth velocity.

**Variables**	**Weight growth velocity**	
	***r***	***p*-value**
Birth weight	−0.162	0.000
Change in *Z*-score of weight	0.688	0.000
GA at birth	−0.018	0.579
Hospital stay	−0.091	0.004
Moderate to severe BPD	−0.002	0.944

### Multiple Linear Regression Analysis

In linear regression analysis, the weight growth velocity was the dependent variable, while the rest of the scores were independent variables (Table 5). The results of multiple linear regression analysis showed that BW, duration of PN, days to regain BW, and change in *Z*-score of weight were the influential factors of the weight growth velocity (Table 5; *p* < 0.05).

**Table 5:** Multivariate linear regression analysis of influencing factors of weight growth velocity.

**Variables**	**Unstandardized coefficients**		**Standardized coefficients**	***t-*value**	***p*-value**	**95.0% CI**
	**β**	***SE***	**β**			
Constant	17.953	1.421		12.634	0.000	(15.165, 20.742)
Birth Weight	−0.004	0.000	−0.339	−12.633	0.000	(−0.005, −0.003)
Duration of PN	−0.016	0.005	−0.063	−2.884	0.004	(−0.026, −0.005)
Loss of birth weight	0.001	0.016	0.002	0.082	0.935	(−0.031, 0.033)
Days to regain BW	0.296	0.017	0.419	16.993	0.000	(0.262, 0.330)
Change in *Z*-score of weight	3.211	0.086	0.768	37.210	0.000	(3.042, 3.380)
GA at birth	0.099	0.051	0.052	1.936	0.053	(−0.001, 0.199)
Energy intake for the first week	0.000	0.001	0.08	0.402	0.688	(−0.001, 0.989)

## Discussion

### The Actuality of Using HMF

The usage rate of HMF is 90–100% in the NICU of developed countries (14–16). In 2013, a study on nutritional care among 25 NICUs in Australia revealed that HMF was administered to infants ranging from 1,250 to 2,500 g in weight, 100% usage (14). A survey conducted in 2015 in the USA exhibited that the use rate of HMF was >90% among premature infants with GA <32 weeks and BW < 1,500 g (16). Expert Consensus on the Use of HMF for Premature Infants in China recommends HMF used for preterm infants with BW < 1,800 g (17). Our study enrolled 985 VPIs with GA < 32 weeks and BW < 1,800 g, of which 847 VPIs (86.0%) were breastfed with HMF, and 14% were not breastfed, indicating a relatively low use rate of HMF in China.

Currently, no consensus exists on the optimal initiation for HMF use. The enteral volume with HMF addition ranged from 50 to 180 ml·kg^−1^·day^−1^ in different NICU (18). Shah et al. compared the weight gain velocity of very low birth weight infants (VLBWI) and discovered that adding HMF at an enteral volume of 20 ml·kg^−1^·day^−1^ resulted in faster weight gain velocity than with 100 ml·kg^−1^·day^−1^, with the corresponding growth velocity of 18.3 and 16.7 g·kg^−1^·day^−1^, respectively (19). The results indicated that early fortification could help promote weight gain. However, Sullivan et al. (20) reported no significant differences in the growth velocity of weight, length, and HC between preterm infants starting fortification at an enteral volume of 40 or 100 ml·kg^−1^·day^−1^. Guidelines for Feeding VLBWI (Canada) recommends to start fortification at an enteral volume of 100 ml·kg^−1^·day^−1^ (4). Moreover, the consensus in China recommends adding HMF at an enteral volume of 50–80 ml·kg^−1^·day^−1^ and achieving full fortification within 3 to 5 days if tolerable (17). The current study demonstrated that HMF was used for 9.0% of VPIs at the early, 25.6% at the middle, and 51.4% at the late fortification group with an enteral volume of ≥100 ml·kg^−1^·day^−1^. The timing of adding HMF was later compared with those in the developed countries. In addition, the time to reach full fortification of the early, the middle, and the late fortification groups were 13.2 ± 11.0, 13.8 ± 11.7, and 12.3 ± 13.0 days, respectively, which suggested no significant differences among the three groups (*p* > 0.05) and thereby indicated a long time from starting HMF until achieving full fortification for Chinese VPIs.

### Effect of HMF on Complications in VPIs

HMF can increase the osmolality of human milk, slow gastric emptying, cause gastric retention, and increase vomiting, thereby presenting NEC risk (2, 21). The fear of feeding intolerance or NEC results in delayed or inadequate fortification feeding. A study of 207 infants of 500–1,250 g reported no significant differences in the incidence of NEC between the groups with HMF added at an enteral volume of 40 and 100 ml·kg^−1^·day^−1^ (20). Another prospective study of 100 VLBWI revealed that starting HMF at an enteral volume of 20 ml·kg^−1^·day^−1^ could improve the early protein intake without increasing feeding intolerance and NEC incidence compared with starting HMF at an enteral volume of 100 ml·kg^−1^·day^−1^ (19). Our study revealed no significant differences in the incidence of feeding intolerance and NEC among the four groups, consistent with previous studies. We thus revealed that adding HMF and different breast milk enhancement strategies did not increase the feeding intolerance and NEC incidence.

Optimizing nutritional support could reduce the incidence and severity of BPD for premature infants and promote the development and injury repair of the lung. Several studies have revealed that insufficient postnatal nutrition supply is an independent risk factor of BPD for premature infants (22). Our study revealed that the early fortification group exhibited the highest incidence of moderate to severe BPD (40.4%), followed by the middle (20.2%) and the late fortification group (15.0%), as explained by the negative correlation between the incidence of BPD and the GA and BW (23). VPIs with lower GA and BW had earlier initiation of adding HMF in this study. In addition, standard fortification strategy (not individualized strategy) was implemented in this study and the time to achieve full fortification was long, leading to insufficient supplement, which could not improve the progression of BPD. Therefore, we recommend to follow expert consensus and use individualized fortification strategies based on nutritional monitoring to reduce the incidence of moderate to severe BPD (24, 25).

There was no significant difference in the incidence of late-onset sepsis in our study. Central venous catheterization is a risk factor of late-onset sepsis for VPIs, and catheter-related bloodstream infection was associated with duration of catheter venous catheterization (26). Our study revealed no significant differences in the time to achieve full enteral feeding and PN duration in four groups. The different initiation of fortification did not have effects on the incidence of late-onset sepsis and duration of PN, which was consistent with the previous studies (27).

### Effect of Fortified Breastfeeding on Growth Status

Despite the overall nutritional improvement of VPIs due to the early parenteral and enteral nutrition support, the incidence of EUGR is still high. EUGR affects growth and development, which are associated with long-term neurocognitive impairment (28). A systematic review demonstrated that preterm infants who received fortified breastfeeding exhibited increased growth velocity of weight, length and HC (29). Our study illustrated that the no-HMF group had the slowest growth velocity and the largest decrease in *Z*-score of weight and length, and a higher incidence of EUGR (61.6%) when compared with those who used HMF, suggesting that the addition of HMF could improve the extrauterine growth status of VPIs.

Our study found that the middle fortification group had the fastest growth velocity and the least dramatic decrease in *Z*-score of weight and length, and the lowest incidence of EUGR (35.7%), which signifies a satisfactory extrauterine growth. In this study, the growth velocity of middle fortification group VPIs was 15.0 ± 2.8 g·kg^−1^·day^−1^ for weight and 1.0 ± 0.4 cm for length. Tudehope et al. (30) proposed that the target growth velocity of infants was 15–20 g·kg^−1^·day^−1^ for weight and 1.0 cm/week for length within 4 weeks after birth. In this study, only the middle fortification group achieved that goal, indicating that the addition of HMF at an enteral volume of 80–100 ml·kg^−1^·day^−1^ was the best practice to promote preterm infant growth. Moreover, no significant differences were noted in the growth velocity and the change in *Z*-score of HC among the four groups, which was consistent with the results reported by Maas et al. (5). Moreover, Roze et al. (31) proposed that the “breastfeeding paradox” is constant in VPIs describing better neurodevelopmental outcomes despite the suboptimal initial weight gain, suggesting better HC growth than overall weight gain in predominantly human milk-fed preterm infants.

### Strengths and Limitations

This study is the first prospective multicenter study on the use of HMF for VPIs in China, covering 28 hospitals, including general hospitals, children's hospitals, and women and children's hospitals across 7 regions of China. All participating NICUs are grade A level III NICUs authorized by the Health Administration of China. We believe that the present study will help neonatologists better understand optimizing the use of HMF in VPIs. However, there still exists some limitations. Firstly, it is an observational study; some potential confounding factors could not be eliminated. Secondly, there may have been some bias and variation, although we used standard techniques to measure the weight, length, and HC. Thirdly, we did not analyze the human milk composition and describe actual enteral and parenteral nutrition received in hospital, so some caution is needed when comparing our results with studies that have reported specific intakes.

## Conclusions

In summary, the usage rate of HMF was found to be relatively low for Chinese VPIs. Fortification often occurred in the late feeding stage, and the time to reach full fortification was long. Adding HMF and different breast milk enhancement strategies did not increase the incidence of feeding intolerance and NEC. The enteral volume of 80–100 ml·kg^−1^·day^−1^ with HMF exhibited better growth in weight and length and lower incidence of EUGR, which suggests that the addition of HMF at that feeding volume might be a best practice toward growth promotion. However, further large sample-sized, prospective, randomized controlled trials are warranted in the future.

## Data Availability Statement

The original contributions presented in the study are included in the article/supplementary material, further inquiries can be directed to the corresponding authors.

## Ethics Statement

The study protocol was approved by the Ethics Committees of Women and Children's Hospital, School of Medicine, Xiamen University (KY-2019-016), recognized by all participating hospitals.

## Author Contributions

XL, RL, WS, and XT conceptualized and designed the study. RL, WS, FW, JM, LL, YC, RZ, XY, YQ, LM, RC, HW, DC, and ZZ carried out the clinical data collection and data analysis. RL and WS wrote the first draft of this manuscript. XL and XT reviewed and revised the manuscript. All authors read and approved the final manuscript.

## Conflict of Interest

The authors declare that the research was conducted in the absence of any commercial or financial relationships that could be construed as a potential conflict of interest.

## Publisher's Note

All claims expressed in this article are solely those of the authors and do not necessarily represent those of their affiliated organizations, or those of the publisher, the editors and the reviewers. Any product that may be evaluated in this article, or claim that may be made by its manufacturer, is not guaranteed or endorsed by the publisher.
